# Extracorporeal Albumin Dialysis (OPAL) as Novel Therapeutic Bridging Option in Posthepatectomy Liver Failure

**DOI:** 10.1111/aor.70187

**Published:** 2026-06-14

**Authors:** Dorian Marckmann, Emely Wack, Luisa Schäfer, H. Christian Hillebrecht, Francesca Reimer, Gabriel Stöger, Klara Krötzsch, Magdalena Menzel, Lampros Kousoulas, Stefan Utzolino, Stefan Fichtner‐Feigl, Dawid L. Staudacher, Frederic Arnold, Thomas Welte, Philipp Holzner, Felix A. Rottmann, Christopher Berlin

**Affiliations:** ^1^ Department of General and Visceral Surgery, Medical Center and Faculty of Medicine University of Freiburg Freiburg Germany; ^2^ Interdisciplinary Medical Intensive Care, Medical Center and Faculty of Medicine University of Freiburg Freiburg Germany; ^3^ Department of Medicine IV – Nephrology and Primary Care, Medical Center and Faculty of Medicine University of Freiburg Freiburg Germany; ^4^ Department of Nephrology University Hospital Zurich Zurich Switzerland; ^5^ Zurich Kidney Center Zurich Switzerland; ^6^ German Cancer Consortium (DKTK) Partner Site Freiburg Germany; ^7^ IMMediate Advanced Clinician Scientist‐Program University of Freiburg Freiburg Germany

## Abstract

**Background:**

Posthepatectomy liver failure (PHLF) remains a major cause of mortality after liver resection, with limited therapeutic options. PHLF can be categorized into primary (small‐for‐size) and secondary forms, the latter resulting from complications such as sepsis or hemorrhage. Extracorporeal albumin dialysis (ECAD), including open albumin dialysis (OPAL), may support hepatic detoxification by removing albumin‐bound toxins. While most studies focus on primary PHLF, data on secondary PHLF are scarce.

**Methods:**

This retrospective cohort study includes all patients receiving OPAL after liver surgery until February 2026 at the Medical Center of the University of Freiburg, Germany. A detailed chart review was performed. The primary endpoint was bilirubin reduction per filter cycle. Survival and changes in laboratory parameters were analyzed as secondary endpoints.

**Results:**

Eighteen patients were included, predominantly with secondary PHLF (15/18, 83.3%). Sepsis was the leading cause of secondary liver failure (8/15, 53.3%), followed by hemorrhage (6/15, 40.0%). Median bilirubin reduction per filter cycle was 1.52 mg/dL (relative reduction 14.64%; *p* < 0.0001), with greater reductions at higher prefilter levels (*p* < 0.0001). Relative reductions were also observed in creatinine (16.84%) and urea (14.75%; both *p* < 0.0001). Platelet count and hemoglobin showed minor decreases, while MELD 3.0 scores improved modestly. Multiple consecutive filters did not increase overall bilirubin reduction. Thirty‐day survival after surgery was 66.7% and 38.9% after OPAL initiation.

**Conclusion:**

OPAL effectively reduces bilirubin levels and improves related parameters in PHLF. It may provide temporary detoxification support, particularly in secondary PHLF. Prospective studies are needed to assess its impact on patient survival.

## Graphical abstract

Extracorporeal albumin dialysis (OPAL) effectively reduces bilirubin and improves other laboratory markers in posthepatectomy liver failure, particularly in secondary forms. It may serve as a bridging therapy to support hepatic recovery in critically ill patients, although prospective studies are needed to confirm its impact on survival.

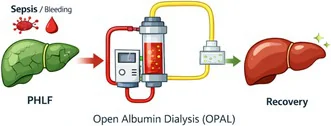

## 1 Introduction

Partial liver resection represents the cornerstone treatment for a range of malignant liver diseases, including hepatocellular carcinoma, cholangiocarcinoma, and colorectal liver metastases, and remains the only potentially curative option for many patients [1, 2, 3].

Despite recent advances in surgical techniques (including robotic approaches and improved assessment of preoperative liver function and future liver remnant volume), these procedures are still associated with considerable morbidity and mortality, reaching up to 30% depending on extent of resection and underlying disease [4, 5, 6, 7, 8, 9]. Posthepatectomy liver failure (PHLF) is the complication associated with the highest mortality, with reported incidences ranging from 0.7% to 9.1%. PHLF can be categorized into primary (small‐for‐size) and secondary liver failure resulting from postoperative complications such as bile leakage, sepsis, or hemorrhage [6, 7, 10, 11, 12].

To date, no universally accepted definition of PHLF exists. However, it is commonly described as impaired excretory and/or synthetic liver function on or after postoperative day (POD) 5, when laboratory parameters usually normalize [6, 13]. Several diagnostic criteria have been proposed, although none have gained universal acceptance. The most widely used definition is the Balzan criterion, which defines primary PHLF as a prothrombin time > 50% combined with serum bilirubin > 50 μmol/L on POD 5 and is associated with a mortality of over 50%. This definition was refined by Mullen et al., who suggested a peak bilirubin threshold of 7 mg/dL (about 120 μmol/L) as a postoperative cutoff with improved sensitivity for predicting mortality (93.3% vs. 50%), also addressing the confounding effect of postoperative fresh frozen plasma administration on prothrombin time interpretation [14, 15]. There is no standardized definition for secondary PHLF, but liver failure attributable to complications occurring after POD 5 has been proposed [6, 12].

Effective treatment options for PHLF remain limited, making prevention crucial. Perioperative administration of steroids might reduce the risk of PHLF [8, 11, 16].

More recently, extracorporeal albumin dialysis (ECAD) has emerged as a therapeutic option for PHLF, potentially bridging detoxification until recovery of liver function, as regeneration and functional gain after resection are expected. ECAD has been shown to reduce levels of creatinine, bilirubin, aminotransferases, and proinflammatory cytokines by removing albumin‐bound toxins, with potential benefits for hepatic encephalopathy and circulatory instability. Several systems are currently available, including Molecular Adsorbent Recirculating System (MARS), Open Albumin Dialysis (OPAL), Advanced Organ Support (ADVOS), Single Pass Albumin Dialysis (SPAD), and the Prometheus system [17, 18]. MARS is the most extensively studied modality and has demonstrated beneficial effects in a prospective study in PHLF, although without a significant survival benefit. OPAL, developed based on the MARS concept, has been reported to achieve superior removal of bilirubin and even ammonia. However, data regarding its use in PHLF are currently lacking [17, 19, 20].

In this retrospective cohort study, we hypothesized that OPAL significantly reduces serum bilirubin levels as a marker of detoxification in posthepatectomy liver failure, with a particular focus on secondary PHLF. Additionally, we aimed to evaluate the effect of OPAL on other relevant laboratory parameters.

## 2 Methods

### 2.1 Data Acquisition

All patients treated with ECAD (i.e., OPAL in all cases) therapy after major liver surgery between July 2023 and February 2026 at the Surgical Intensive Care Unit (ICU) of the Medical Center of the University of Freiburg, Germany were included in this registry. Analysis followed the design of a retrospective cohort study. The ethics committee of the University of Freiburg (ETK‐FR: 25‐1295_1‐S1‐retro) approved this registry. The RECORD statement [21] was adhered to when checking data for plausibility and presentation follows the STROBE guidelines [22]. Inclusion criteria were an age of ≥ 18 years at the day of liver surgery and OPAL for ≥ 1 cycle. Patient identification was achieved via a computerized search in our hospital database for all patients receiving OPAL. Individual patient files including charts and discharge letters were analyzed manually on a case‐by‐case basis.

Primary endpoint was bilirubin reduction per OPAL filter treatment. Secondary endpoints were changes of creatinine, urea, leucocyte count, platelet count, hemoglobin, alanine aminotransferase (ALT), and MELD 3.0 scores (i.e., without albumin), as well as the bilirubin reduction after multiple OPAL filters. As OPAL also treats kidney failure, chronic kidney disease (CKD) was assessed as a baseline parameter.

For calculation of MELD 3.0 scores, the creatinine value was set to 3 mg/dL for all instances according to the official recommendation for patients undergoing dialysis, since kidney function is not reliably assessable by creatinine‐based eGFR calculation during OPAL. Since albumin was not frequently assessed, MELD 3.0 was calculated without albumin [23, 24, 25].

PHLF was defined according to Mullen et al.'s criterion as peak bilirubin > 7 mg/dL on POD 5 or later due to possible confounding of the Balzan criterion by volume therapy with fresh frozen plasma in the postoperative period [15]. PHLF was defined as secondary if peak bilirubin > 7 mg/dL occurred after POD 5 combined with a relevant complication (Clavien‐Dindo ≥ IV) [26]. If certain laboratory values were not present daily (e.g., values to calculate MELD 3.0 score), analyses were only performed for time spans with the respective parameters.

One patient (patient 16, Tables 1 and 2) did not meet the peak bilirubin > 7 mg/dL criterion due to early OPAL initiation but fulfilled the Balzan criterion for PHLF on day 5 and was therefore included and managed the same as patients meeting the peak bilirubin > 7 mg/dL criterion [14]. Two patients (patient 16 and 18, Tables 1 and 2) met the criteria for PHLF on POD 5 and were therefore classified as primary PHLF, although evident secondary causes were present, as detailed in Section 3.

**TABLE 1:** Baseline characteristics and surgical treatment.

Pt	Dx	Age	UICC grade	Neoadj. Cx	CKD	ISS/PVE	Surgery	Res. segm. (*n*)
1	iCCA	72	IIIB	1	0	0	HH left + I	5
2	iCCA	70	I	0	0	0	HH left + I + atypical IVa + b	7
3	iCCA	67	II	0	0	0	HH right	4
4	iCCA	75	IB	0	0	1	Extended HH right + I	7
5	iCCA	71	IV	1	0	0	Extended HH right	6
6	eCCA	57	IV	0	0	0	HH right + IVb	5
7	eCCA	67	IIIB	1	0	1	HH right + I + atypical IVb	6
8	eCCA	82	II	0	0	0	MH	5
9	eCCA	54	II	0	0	0	MH	4
10	eCCA	66	IIIC	0	0	0	Extended HH left	6
11	HCC/iCCA	67	—	0	0	0	HH right	4
12	HCC‐LTx	65	IIIA	0	1	0	IVb + V + atypical III	3
13	CRLM	73	IVA	1	0	0	MH + atypical II, III, VI, VII	8
14	GBC	66	IIIB	0	0	1	Extended HH right + atypical III	7
15	AE	47	—	—	1	0	HH right	4
16	CLA/HI	72	—	—	0	0	HH right	4
17	CLA‐LTx	62	—	—	0	0	HH right	4
18	CL	67	—	—	0	1	HH right	4
	CCA 61.1%	67 (65–72)		28.6%	11.1%	22.2%		5 (4–6)

*Note:* 1 = yes, 0 = no. Bottom line data are presented as median (IQR) or number of patients (percentage of group). Percentages are calculated based on available data; missing values are indicated as “—”.

Abbreviations: AE, alveolar echinococcosis; CKD, chronic kidney disease; CL, cholelithiasis; CLA, cholangitic liver abscesses; CRLM, colorectal liver metastases; Cx, chemotherapy; Dx, diagnosis; eCCA, extrahepatic cholangiocarcinoma; GBC, gallbladder carcinoma; HCC, hepatocellular carcinoma; HH, hemihepatectomy; HI, hepatic ischemia; iCCA, intrahepatic cholangiocarcinoma; ISS, in situ‐split; LTx, liver transplantation; MH, mesohepatectomy; Neoadj. Cx, neoadjuvant chemotherapy; PC, pancreatic carcinoma; Pt, patient; PVE, portal vein embolization; Res. segm., resected segments.

**TABLE 2:** Liver failure, OPAL treatment, and survival.

Pt	LF	Reason for LF	PHLF (POD)	OPAL filters (*n*)	Bilirubin OPAL start (mg/dL)	OPAL start (POD)	30‐day surv.	90‐day surv.
1	Sec.	Sepsis (cholangitis)	34	5	23.09	78	1	1
2	Prim.	—	5	38	16.67	8	1	1
3	Sec.	Sepsis (cholangitis)	30	11	9.83	31	1	0
4	Sec.	Sepsis (cholangitis)	8	3	16.19	21	0	0
5	Sec.	Sepsis (pneumonia)	11	2	10.17	22	1	0
6	Sec.	Portal vein thrombosis	6	6	7.36	6	0	0
7	Sec.	Sepsis (anastomotic leak)	12	4	15.66	21	0	0
8	Sec.	Sepsis (Candida infection)	7	2	6.93	21	0	0
9	Sec.	Hemorrhage	25	27	5.02	12	1	1
10	Sec.	Hemorrhage	19	11	11.15	37	1	1
11	Sec.	Hemorrhage	7	10	7.78	7	1	0
12	Sec.	Hemorrhage	37	3	7.39	37	1	0
13	Sec.	Sepsis (cholangitis)	26	1	14.72	32	1	0
14	Sec.	Hemorrhage, colon ischemia	9	1	9.46	15	0	0
15	Sec.	Sepsis (cholangitis)	29	6	22.53	62	1	1
16	Prim.	Sepsis (cholangitis)	5	3	5	8	0	0
17	Sec.	Hemorrhage	18	2	11.39	27	1	—
18	Prim.	Sepsis (cholangitis)	5	16	3.93	0	1	—
	Prim. 16.7%	Sepsis 55.6%	11.5 (7–26)	4.5 (2–11)	10 (7.4–15.7)	21 (8–32)	66.7%	31.3%

*Note:* 1 = yes, 0 = no. Bottom line data are presented as median (IQR) or *n* (%). Percentages are calculated based on available data; missing values are indicated as “—”.

Abbreviations: LF, liver failure; OPAL, open albumin dialysis; PHLF, posthepatectomy liver failure; POD, postoperative day; Prim., primary; Pt, patient; Sec., Secondary; surv., survival.

### 2.2 Local ECAD Standards

As ECAD‐variant of choice our center currently only deploys OPAL [20]. Therapy is available at our center nearly 24/7 but limited by availability of a dialysis nurse experienced in assembling this highly complex system: Basis of the technical setup is a conventional citrate dialysis circuit modified to include one conventional dialysis filter indirectly connecting blood (1) and albumin circuit (2) and a second conventional dialysis filter connecting albumin circuit (2) and standard dialysate (3). The central albumin circuit contains a single albumin filter (Hepalbin‐MaxiCycler, Albutec GmbH, Rostock, Germany) enabling extracorporeal removal of albumin‐bound toxins via continuous albumin regeneration. This setup enables regular dialysis for up to 3 days, but Albutec guarantees albumin purification only up to the first 24 h. Thus, the entire system including two dialysis filters and the albumin filter was exchanged if treatment with a new Hepalbin filter was indicated.

Of note, the Hepalbin‐MaxiCycler can be considered an adsorber on a technical level but as it is generally described as a “filter” by the manufacturer we opted to maintain their terminology to avoid further confusion.

Indication follows a case‐by‐case decision by the clinician at the bedside, normally during but not limited to morning rounds with the senior physicians of the ICU. Accordingly, most OPAL therapies were initiated during daytime. Discontinuation of OPAL was also determined on a case‐by‐case basis according to clinical and laboratory improvement or deterioration. If anticoagulation was required (e.g., due to thrombosis or vascular reconstruction), a continuous infusion of unfractionated heparin was used. Typical parameters to initiate OPAL are elevated serum bilirubin, impaired coagulation parameters in combination with cardiorespiratory instability. Until now, no uniform cutoff values for initiation or discontinuation of OPAL are applied.

### 2.3 Statistical Analysis

As laboratory results were generally measured once per calendar day (generally at 6 am), changes in these values after the initiation of OPAL were evaluated every 24 h. These changes were only evaluated when OPAL operated > 12/24 h between blood sampling (at least half of the day from 6 am to 6 am the next day). We considered filters that were changed in < 24 h after completion of the previous treatment session to be in close succession and used for comparison of single versus multiple filters. For this comparison, values were taken from the last blood sample before the first filter and compared with the last sample during OPAL treatment or the first blood sample after treatment completion.

Wilcoxon's signed‐rank test was used for analyzing paired data and Mann–Whitney *U* test for unpaired data. The association between prefilter values and absolute reduction was assessed using Spearman's rank correlation. Linear regression was applied for trend visualization. Data are presented as median (interquartile range) or number of patients (percentage of group). *p* values < 0.05 were considered to be statistically significant. GraphPad Prism 11 (GraphPad, San Diego, CA, USA) was used for statistical analysis and graph creation.

## 3 Results

### 3.1 Baseline and Surgical Treatment

A total of 18 patients were treated with OPAL between July 2023 and February 2026. Median age was 67 years and 7/18 (38.9%) were female. Four patients (22.2%) underwent surgical treatment for nonmalignant disease. Fourteen patients (77.8%) received therapy for malignant disease, out of which 10 were suffering from cholangiocarcinoma (CCA). In one patient, differentiation between hepatocellular carcinoma (HCC) and combined HCC‐CCA was not possible (patient 11). Union for International Cancer Control (UICC) grades ranged from I to IV, and four patients received neoadjuvant therapy. Only two patients, one with alveolar echinococcosis and one with HCC after liver transplantation, suffered from CKD before surgery. Two patients had undergone liver transplantation prior to the operation (patient 12 and 17). A median of five liver segments (anatomically or partial/atypically) were resected during surgery.

In total, four patients received either preresectional portal vein embolization or a surgical two‐step procedure (in situ‐split). The patients with nonmalignant disease were operated on for various reasons. Patient 16 developed cholangitis and subsequent occlusion of the right hepatic artery 2 months after pancreaticoduodenectomy for chronic pancreatitis. Patient 17 was suffering from biliary stenosis after liver transplantation with recurrent cholangitis and developed a large intrahepatic abscess. Patient 18 underwent elective cholecystectomy at a peripheral hospital with accidental occlusion of the right hepatic duct without the possibility of reconstruction. Patients 17 and 18 still underwent intensive care treatment when we stopped study inclusion. Table 1 summarizes baseline characteristics and surgical treatment.

### 3.2 Liver Failure, OPAL Treatment, and Survival

Only three patients (16.7%) suffered from primary PHLF by definition. Of these, two were already suffering from sepsis due to cholangitis (patient 16 with cholangitic abscess after pancreaticoduodenectomy and patient 18 with biliary leakage after occlusion of the right hepatic duct in elective cholecystectomy) when surgery was performed. Therefore, alternative secondary reasons for these cases of primary PHLF seem plausible. Still, most patients developed secondary PHLF, with sepsis (*n* = 8/15; 53.3%) being the predominant cause, most commonly due to cholangitis. The second most common cause was hemorrhage (*n* = 6/15; 40.0%). On median, PHLF occurred on day 11.5 after surgery and OPAL was initiated on day 21. A median of 4.5 filters were used per patient. The median bilirubin serum level at OPAL initiation was 10 mg/dL. 12/18 patients (66.7%) reached 30‐day survival after surgery, 5/16 patients (31.3%) survived at least 90 days. Table 2 summarizes liver failure, OPAL treatment and survival for each patient.

Interestingly, if Balzan‐criteria were applied, still most of the patients (*n* = 16/18; 88.9%) fulfilled the definition at some point with a similar median of the POD when the definition was met (POD 11 [IQR 6–26] for Balzan criterion vs. POD 11.5 [IQR 7–26] for the criterion by Mullen et al.). One patient only fulfilled the Balzan criterion on POD 5 and never fulfilled the peak bilirubin criterion due to early OPAL initiation and reduction of bilirubin serum levels.

### 3.3 OPAL Analysis

**FIGURE 1:**
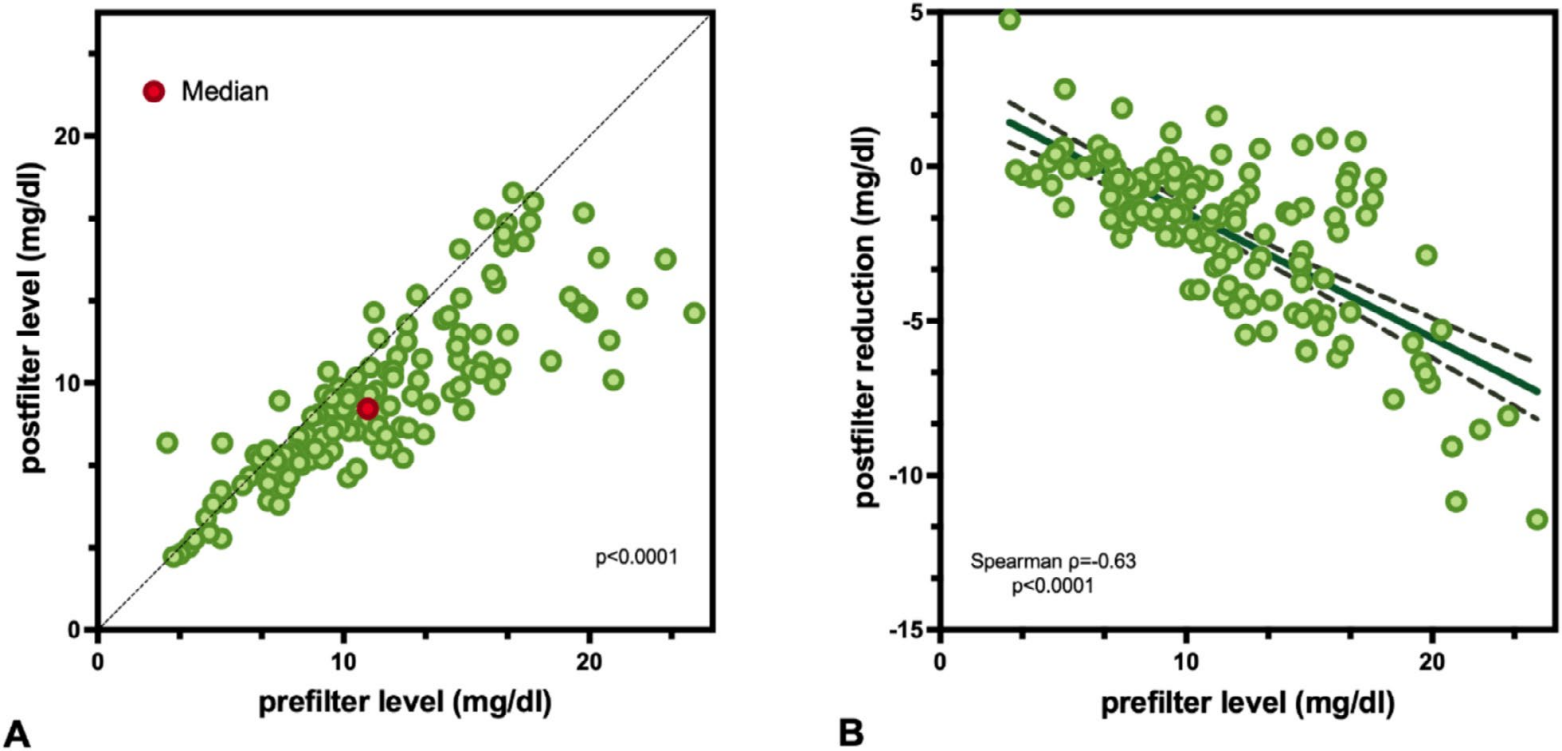
Bilirubin reduction during OPAL with filter activity > 12 h per 24 h. Bilirubin reduction across all filters running > 12 h per 24 h. (A) Scatter plot of prefilter versus postfilter bilirubin levels; the dashed line indicates equality (no change). Differences were analyzed using the Wilcoxon's signed‐rank test. (B) Association between prefilter bilirubin levels and absolute bilirubin reduction; the line represents linear regression. Correlation was assessed using Spearman's rank correlation. [Color figure can be viewed at wileyonlinelibrary.com]

**FIGURE 2:**
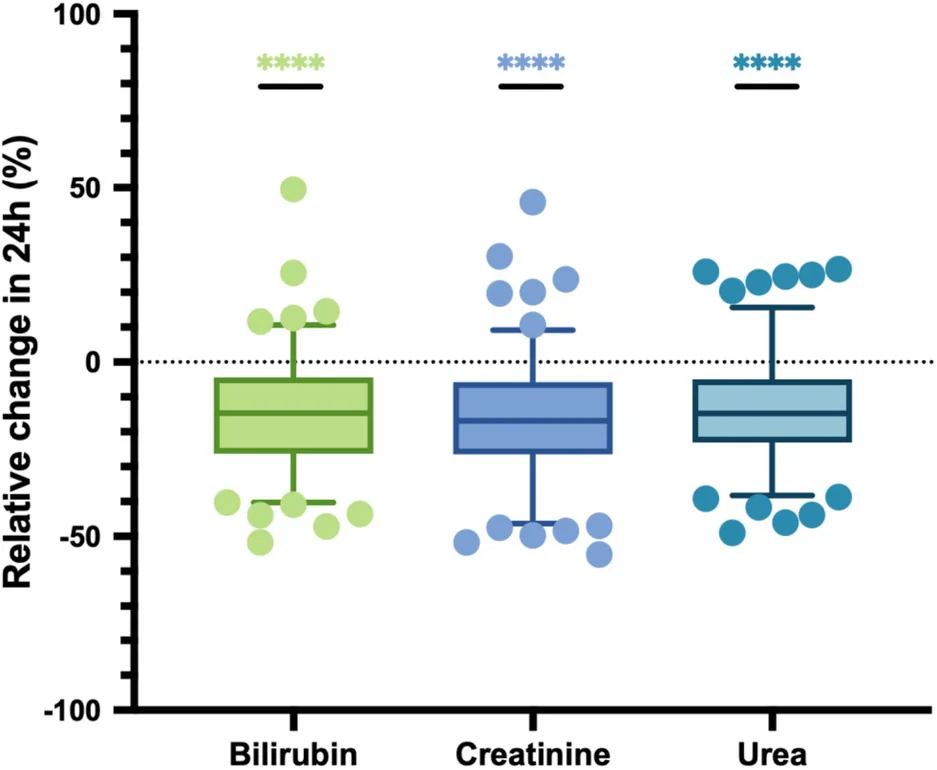
Relative change in serum bilirubin levels, creatinine, and urea during OPAL with filter activity > 12 h per 24 h. Significant reductions are indicated by asterisks. Boxes represent median and interquartile range (IQR), whiskers indicate the 5th–95th percentile. Differences were analyzed using the Wilcoxon signed‐rank test. Significance levels are indicated as follows: ****p < 0.0001. [Color figure can be viewed at wileyonlinelibrary.com]

**FIGURE 3:**
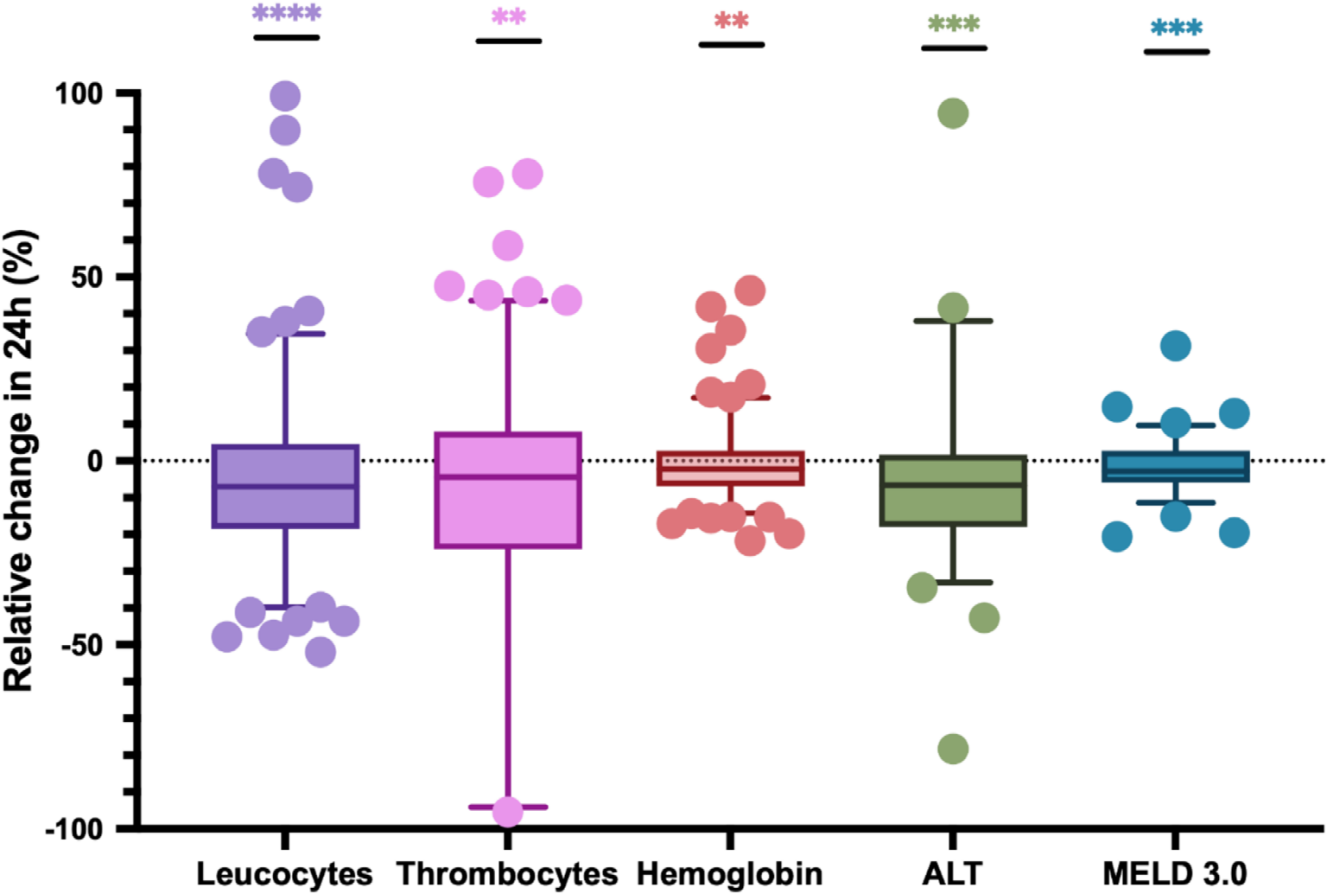
Relative change in leucocyte count, platelet count, hemoglobin, alanine aminotransferase (ALT), and MELD 3.0 score during OPAL with filter activity > 12 h per 24 h. Significant reductions are indicated by asterisks. Boxes represent median and interquartile range (IQR), whiskers indicate the 5th–95th percentile. Differences were analyzed using the Wilcoxon signed‐rank test. Significance levels are indicated as follows: **p < 0.01, ***p < 0.001, ****p < 0.0001. [Color figure can be viewed at wileyonlinelibrary.com]

**FIGURE 4:**
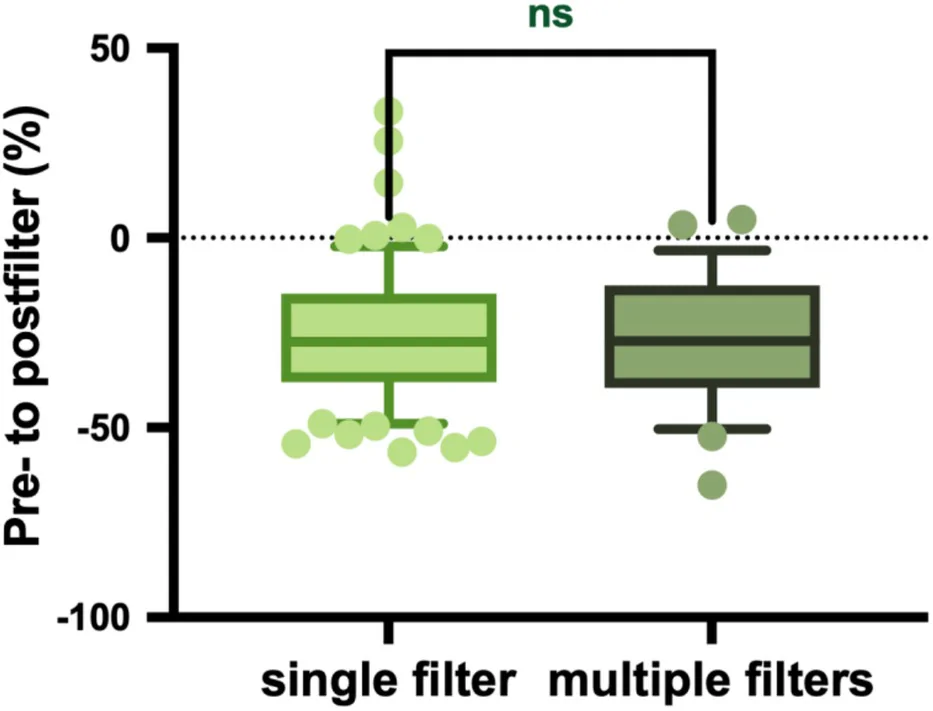
Comparison of relative change in serum bilirubin levels between single and multiple consecutive OPAL filters. Nonsignificant differences are indicated by “ns.” Boxes represent median and interquartile range (IQR); whiskers indicate the 5th–95th percentile. Differences were analyzed using the Mann–Whitney *U* test. [Color figure can be viewed at wileyonlinelibrary.com]

**FIGURE 5:**
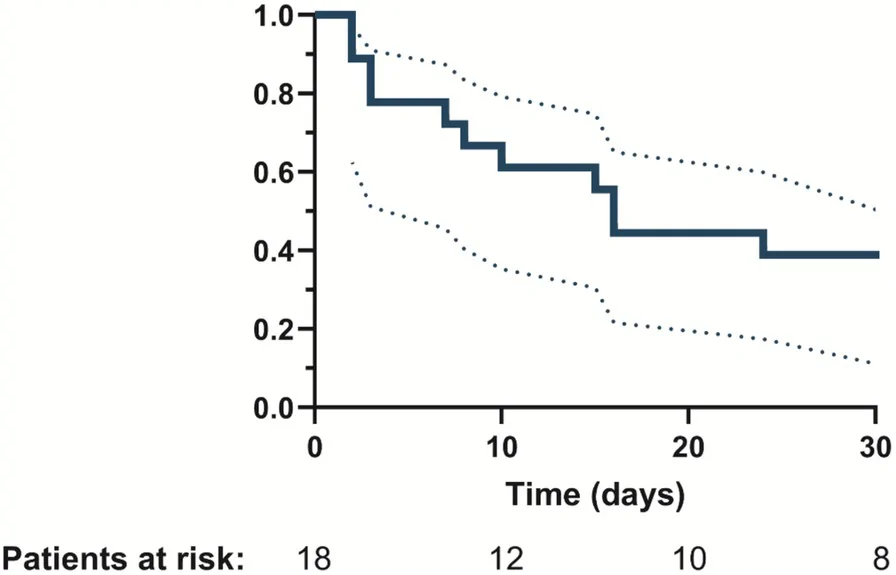
Kaplan–Meier curve of 30‐day survival after OPAL initiation (*n* = 18). The dotted lines represent the 95% confidence intervals and the number of patients at risk is displayed below the *x*‐axis. [Color figure can be viewed at wileyonlinelibrary.com]

Bilirubin reduction during OPAL treatment operating for a minimum of 12 out of 24 h was measured at a median of 1.52 (0.37–3.31) mg/dL (*p* < 0.0001, Figure 1A). As expected, absolute reduction increased with the prefilter bilirubin serum level (*p* < 0.0001, Figure 1B). Median relative reductions after at least 12 h of OPAL filter ranged from 14.64% in bilirubin (*p* < 0.0001), 16.84% in creatinine (*p* < 0.0001) to 14.75% in urea (*p* < 0.0001; Figure 2). Additionally, we noted changes in several other laboratory values: platelet count and hemoglobin decreased slightly after OPAL treatment with a median reduction of 7.01% (*p* > 0.0001) and 2.17% (*p* < 0.01) respectively. In contrast, we observed improvement of leucocyte counts with a median reduction of 4.40% (*p* < 0.01) and reduction of alanine aminotransferase (6.67%; *p* < 0.001) by OPAL. A slight decrease in MELD 3.0 scores (median of 1 point; *p* < 0.001) was seen even though creatinine level was set to 3 mg/dL for calculation due to ongoing dialysis (Figure 3). Of note, the use of multiple consecutive OPAL filters (direct change of filters after completion of a treatment session) did not result in a significantly larger reduction of bilirubin levels after complete treatment cycles compared to treatments with one filter only (Figure 4). At most, six filters were used in close succession. Thirty‐day survival was achieved in 7/18 (38.9%) patients after initiation of OPAL (Figure 5).

We observed stable bilirubin serum levels for over 24 h after discontinuation of OPAL when compared to the last measurement during therapy (median reduction −0.27 mg/dL), but measurements were only available for 5 of 18 patients. Afterwards, a slight increase was noted, but with values from only three patients (Table S1).

## 4 Discussion

Our study demonstrates that OPAL treatment is effective in reducing bilirubin, creatinine, and urea levels in this highly challenging postoperative patient population. Interestingly, MELD 3.0 scores also showed a statistically significant, albeit modest, improvement. Previous studies reporting improvements in MELD scores calculated the score using creatinine values measured during ongoing ECAD therapy, thereby limiting the ability to distinguish true effects on hepatic synthesis and detoxification from dialysis‐related changes [17, 20]. To address this limitation, we set all creatinine values used for MELD 3.0 calculation to 3 mg/dL, ensuring that the observed changes reflect improvements in nonrenal parameters [25].

Further potentially beneficial effects are the slight reduction of leucocyte counts and alanine aminotransferase levels. On the other hand, the decrease of platelet counts and hemoglobin (both with only minor changes) is a potential disadvantage. Both changes have been described in the literature previously [18, 20].

PHLF is a feared complication with high mortality, yet no causal therapy exists to date [8, 10]. Prevention remains key, with perioperative steroids being the only medication shown to be beneficial, making preoperative evaluation of liver function and future remnant size the most important factors. Once PHLF develops, treatment is mainly supportive intensive care, while specific pharmacological therapies such as N‐acetylcysteine or omega‐3 fatty acids have not proven effective [8, 16]. Ursodeoxycholic may have beneficial effects but has not been specifically evaluated in this setting [27]. Finally, liver transplantation may represent a rescue option in selected cases but it remains a high mortality procedure and definitive criteria for patient selection are unavailable [8]. ECAD might be a bridging option in PHLF but data showing survival benefits are missing so far [28].

The use of ECAD for secondary PHLF has not been systematically evaluated to date, as most studies focus on selected patients with primary PHLF treated with MARS. Only a few cases of MARS treatment for secondary PHLF have been reported, and the 90‐day mortality ranged from 67% to 100%. Overall mortality was high, with some differences due to patient selection [19, 28, 29, 30, 31, 32]. Our cohort represents patients undergoing complex and extensive resections with severe, potentially lethal complications, in whom OPAL was applied as rescue therapy. Accordingly, the expected mortality in this population is inherently high, even without secondary complications, and differs substantially from cohorts undergoing less extensive resections. The 30‐day survival rates of 66.7% and 38.9% after initiation of the first filter for patients with predominantly secondary PHLF are broadly consistent with the limited available literature (85.7% in seven patients with secondary PHLF across two studies). Notably, long‐term outcomes appear more favorable in our cohort, with a 90‐day survival rate of 31.2% compared to 14% previously reported. The high proportion of patients with cholangiocarcinoma in our study further contributes to the elevated risk of postoperative morbidity and mortality [2, 19, 28].

To our knowledge, OPAL has not previously been evaluated for PHLF, although it was shown to provide enhanced removal of ammonia and other toxins through addition of a charcoal adsorber and a more efficient albumin regeneration [17, 20]. In particular, the reduction of toxic bile acids may support liver regeneration and decrease side effects of excretion failure like toxic cardiomyopathy. Robust studies demonstrating relevant reduction of toxic bile acids during ECAD therapy are lacking. In clinical practice, serum bilirubin is commonly used as a surrogate for liver detoxification, despite the absence of clear evidence for direct toxic effects of bilirubin itself [33, 34, 35, 36].

Moreover, ECAD therapy has been reported to improve hemodynamic instability and hepatic encephalopathy [18]. In this context, the use of OPAL in patients with secondary PHLF, particularly in the presence of potentially treatable complications such as hemorrhage or sepsis, appears to be a rational strategy to bridge impaired liver detoxification until recovery. The distribution of complications in our cohort is consistent with reports in the literature, with abdominal sepsis and hemorrhage representing the most frequent triggers for secondary PHLF [5, 10, 12]. Of course, the usage of OPAL in PHLF has important limitations that must be carefully considered when indicating this rescue therapy. Besides the lack of robust evidence (especially on survival and other long‐term parameters), OPAL requires substantial technical expertise and considerable financial resources [17, 20]. Furthermore, potential adverse effects on coagulation parameters must be considered, as reflected by the significant decrease in platelet count (Figure 3). Table 3 summarizes potential advantages and disadvantages of OPAL usage in PHLF.

**TABLE 3:** Potential advantages and limitations of OPAL in the treatment for PHLF.

Advantages of OPAL	Disadvantages/limitations of OPAL
Effective reduction of bilirubin and albumin‐bound toxins	No proven survival benefits as of 2026
Significant ammonia reduction, potentially superior to MARS	Evidence limited to retrospective and small cohort studies, no data on PHLF so far
Continuous albumin regeneration via Hepalbin Cluster (less albumin consumption)	Technically complex setup requiring trained personnel (i.e., limited availability)
Treatment duration up to 24 h	Risk of bleeding by thrombocytopenia and coagulation disturbances
Simultaneous removal of water‐soluble toxins (i.e., dialysis)	High treatment costs and resource utilization
Potential bridge to liver recovery	Lack of standardized indications and patient selection
Possible positive cardiovascular effects	

As a potential therapeutic rationale, OPAL may provide temporary detoxification support in selected patients, potentially allowing time to address the underlying cause of secondary PHLF. Prospective studies are required to determine whether this approach translates into improved survival.

A recurrent challenge in the field is the lack of a uniform definition of primary and secondary PHLF. Although the International Study Group of Liver Surgery proposed a consensus definition, it remains relatively nonspecific, describing liver failure primarily as deterioration of postoperative laboratory values compared to preoperative levels, without clearly defined cutoff thresholds. Furthermore, secondary liver failure was not specifically addressed [6]. This likely reflects PHLF being a clinical continuum without a clear diagnostic threshold. The most established definition remains the 50–50 Balzan criterion, which incorporates serum bilirubin and prothrombin time and therefore reflects hepatic synthetic and detoxification function. Mullen et al. demonstrated the limited sensitivity of this definition in predicting mortality and proposed a peak bilirubin > 7 mg/dL as an alternative criterion that was also applied for this study [14, 15]. This approach also mitigates the confounding effect of postoperative therapy with fresh frozen plasma administration on coagulation parameters as indicators of synthetic function. Most previous studies focused on primary PHLF and robust data on the pathogenesis and definition of secondary PHLF remain scarce. Overall, establishing clear definitions for primary and secondary PHLF would greatly facilitate the design and interpretation of future prospective studies.

The retrospective and single‐center design and relatively small cohort size reflect the rarity of these severe complications and limit the generalizability of our findings. The heterogeneous patient population, extent of liver resection, and cause of secondary PHLF further limits comparability between patients. A standardized threshold for initiation and discontinuation of ECAD therapy is lacking. The lack of a control group prevented the identification of therapy‐specific complications, for example, further disruption of coagulation parameters. Furthermore, surrogate laboratory parameters such as bilirubin reduction do not necessarily translate into clinically meaningful benefit or improved survival.

The timing of ECAD initiation may be crucial, as previous studies suggest that earlier treatment in primary PHLF may improve outcome [28]. In our cohort, OPAL was often initiated several days after PHLF‐criteria were met (Table 2). This question should be addressed in future prospective trials evaluating OPAL as a therapeutic option in PHLF.

## 5 Conclusion

OPAL represents a potential therapeutic option for PHLF, a severe and potentially lethal complication following liver resection, for which no standardized treatment recommendations currently exist. Our findings demonstrate that OPAL efficiently reduces bilirubin, urea, and creatinine levels in primary and secondary PHLF and is associated with modest improvements in MELD 3.0 scores. As therapeutic rationale, OPAL may support hepatic detoxification until possible recovery of liver function, particularly in secondary failure. Prospective studies are required to further define the potential benefits, limitations, and adverse effects of OPAL and to establish standardized criteria and protocols for its application in both primary and secondary PHLF.

## Author Contributions

D.M. contributed to study design, supervision, data acquisition, data analysis and interpretation, patient care, manuscript writing and revision. E.W. contributed to data acquisition, data analysis and interpretation and manuscript revision. L.S., F.R., G.S., K.K., M.M., L.K., P.H., H.C.H, S.U. contributed to patient care, follow up and manuscript revision. S.F.‐F. contributed to manuscript revision, resources and to patient care. D.L.S. contributed to study design, data analysis and interpretation and manuscript revision. F.A. contributed to patient care, data analysis and interpretation and manuscript revision. T.W. contributed to data analysis and interpretation and manuscript revision. F.A.R. contributed to study design, supervision, data acquisition, data analysis and interpretation, patient care, manuscript writing and revision. C.B. contributed resources, to supervision, data interpretation and manuscript revision.

## Funding

This work was supported by Medizinische Fakultät der Albert‐Ludwigs‐Universität Freiburg, Bundesministerium für Bildung, Wissenschaft, Forschung und Technologie (01EO2103), and Universität Zürich.

## Conflicts of Interest

D.M., E.W., L.S., F.R., G.S., M.M., L.K., S.U., S.F.‐F., F.A., T.W., P.H., F.A.R., and C.B. declare no conflicts of interest. D.L.S. received lecture honoraria or travel support from Abiomed, AstraZeneca, Dahlhausen, Getinge, Medtronic, Orion Pharma, and was part of a dual lumen advisory board by Medtronic.

## Supporting information


**Table S1:** Course of bilirubin after discontinuation of OPAL. h, hours; Pt, patient. ~ indicates the last laboratory testing during OPAL running time, 0 h indicates the first laboratory testing after discontinuation of OPAL, Δ indicates the change compared to the value during OPAL running time, − indicates the absence of data because patients deceased, are still under OPAL treatment at the time of assessment or laboratory values are missing at the respective time points.

## Data Availability

The data that support the findings of this study are available from the corresponding author upon reasonable request.

## References

[1] J. Bruix and M. Sherman , “Management of Hepatocellular Carcinoma: An Update,” Hepatology 53 (2011): 1020–1022, 10.1002/hep.24199. 21374666 PMC3084991

[2] U. Cillo , C. Fondevila , M. Donadon , et al., “Surgery for Cholangiocarcinoma,” Liver International 39 (2019): 143–155, 10.1111/liv.14089. 30843343 PMC6563077

[3] L. Pérez‐Santiago , D. Huntley Pascual , J. S. Sánchez Lara , M. Huerta , and D. Dorcaratto , “How to Integrate Surgery Into the Multidisciplinary Treatment of Liver‐Only Metastatic Colorectal Cancer,” Cancers 18 (2026): 489, 10.3390/cancers18030489. 41681961 PMC12896976

[4] J. Belghiti , K. Hiramatsu , S. Benoist , P. P. Massault , A. Sauvanet , and O. Farges , “Seven Hundred Forty‐Seven Hepatectomies in the 1990s: An Update to Evaluate the Actual Risk of Liver Resection,” Journal of the American College of Surgeons 191 (2000): 38–46, 10.1016/S1072-7515(00)00261-1. 10898182

[5] Y. Midorikawa , K. Kubota , T. Takayama , et al., “A Comparative Study of Postoperative Complications After Hepatectomy in Patients With and Without Chronic Liver Disease,” Surgery 126 (1999): 484–491, 10.1016/S0039-6060(99)70089-9. 10486600

[6] N. N. Rahbari , O. J. Garden , R. Padbury , et al., “Posthepatectomy Liver Failure: A Definition and Grading by the International Study Group of Liver Surgery (ISGLS),” Surgery 149 (2011): 713–724, 10.1016/j.surg.2010.10.001. 21236455

[7] J. A. Søreide and R. Deshpande , “Post Hepatectomy Liver Failure (PHLF)—Recent Advances in Prevention and Clinical Management,” European Journal of Surgical Oncology 47 (2021): 216–224, 10.1016/j.ejso.2020.09.001. 32943278

[8] K. M. C. van Mierlo , F. G. Schaap , C. H. C. Dejong , and S. W. M. Olde Damink , “Liver Resection for Cancer: New Developments in Prediction, Prevention and Management of Postresectional Liver Failure,” Journal of Hepatology 65 (2016): 1217–1231, 10.1016/j.jhep.2016.06.006. 27312944

[9] Z. Wen , P. Hao , and L. Yang , “Comparison of Efficacy Between Robotic and Open Hepatectomy: A Systematic Review and Meta‐Analysis of Propensity Score‐Matched Studies,” Journal of Robotic Surgery 19 (2025): 162, 10.1007/s11701-025-02326-0. 40237917

[10] R. Kauffmann and Y. Fong , “Post‐Hepatectomy Liver Failure,” Hepatobiliary Surgery and Nutrition 3 (2014): 238–246, 10.3978/j.issn.2304-3881.2014.09.01. 25392835 PMC4207837

[11] M. A. J. Van Den Broek , S. W. M. Olde Damink , C. H. C. Dejong , et al., “Liver Failure After Partial Hepatic Resection: Definition, Pathophysiology, Risk Factors and Treatment,” Liver International 28 (2008): 767–780, 10.1111/j.1478-3231.2008.01777.x. 18647141

[12] A. M. van Keulen , S. Buettner , M. G. Besselink , et al., “Primary and Secondary Liver Failure After Major Liver Resection for Perihilar Cholangiocarcinoma,” Surgery 170 (2021): 1024–1030, 10.1016/j.surg.2021.04.013. 34020794

[13] C. Reissfelder , N. N. Rahbari , M. Koch , et al., “Postoperative Course and Clinical Significance of Biochemical Blood Tests Following Hepatic Resection,” British Journal of Surgery 98 (2011): 836–844, 10.1002/bjs.7459. 21456090

[14] S. Balzan , J. Belghiti , O. Farges , et al., “The ‘50‐50 Criteria’ on Postoperative Day 5,” Annals of Surgery 242 (2005): 824–829, 10.1097/01.sla.0000189131.90876.9e. 16327492 PMC1409891

[15] J. T. Mullen , D. Ribero , S. K. Reddy , et al., “Hepatic Insufficiency and Mortality in 1,059 Noncirrhotic Patients Undergoing Major Hepatectomy,” Journal of the American College of Surgeons 204 (2007): 854–862, 10.1016/j.jamcollsurg.2006.12.032. 17481498

[16] A. Gerritsen , M. T. de Boer , C. I. Buis , et al., “Pharmacological Management for Prevention and Treatment of Posthepatectomy Liver Failure,” Digestive Surgery 42 (2025): 1–11, 10.1159/000548937. 41105553 PMC12688328

[17] J. Friebus‐Kardash , A. Louzi , A. Kribben , et al., “Comparison of Open Albumin Dialysis (OPAL) With Prometheus Fractionated Plasma Separation and Adsorption (FPSA) and Standard Medical Treatment for Acute‐on‐Chronic Liver Failure,” Artificial Organs 49 (2025): 997–1011, 10.1111/aor.14977. 39995389 PMC12120814

[18] F. Saliba , R. Bañares , F. S. Larsen , et al., “Artificial Liver Support in Patients With Liver Failure: A Modified DELPHI Consensus of International Experts,” Intensive Care Medicine 48 (2022): 1352–1367, 10.1007/s00134-022-06802-1. 36066598

[19] S. Gilg , E. Sparrelid , J. Engstrand , et al., “Molecular Adsorbent Recirculating System Treatment in Patients With Post‐Hepatectomy Liver Failure: Long‐Term Results of a Pilot Study,” Scandinavian Journal of Surgery 111 (2022): 48–55, 10.1177/14574969221112224. 36000747

[20] O. Sommerfeld , C. Neumann , J. Becker , et al., “Extracorporeal Albumin Dialysis in Critically Ill Patients With Liver Failure: Comparison of Four Different Devices—A Retrospective Analysis,” International Journal of Artificial Organs 46 (2023): 481–491, 10.1177/03913988231191952. 37609875 PMC10483887

[21] E. I. Benchimol , L. Smeeth , A. Guttmann , et al., “The REporting of Studies Conducted Using Observational Routinely‐Collected Health Data (RECORD) Statement,” PLoS Medicine 12 (2015): e1001885, 10.1371/journal.pmed.1001885. 26440803 PMC4595218

[22] E. von Elm , D. G. Altman , M. Egger , et al., “The Strengthening the Reporting of Observational Studies in Epidemiology (STROBE) Statement: Guidelines for Reporting Observational Studies,” Journal of Clinical Epidemiology 61 (2008): 344–349, 10.1016/j.jclinepi.2007.11.008. 18313558

[23] W. R. Kim , A. Mannalithara , J. K. Heimbach , et al., “MELD 3.0: The Model for End‐Stage Liver Disease Updated for the Modern Era,” Gastroenterology 161 (2021): 1887–1895.e4, 10.1053/j.gastro.2021.08.050. 34481845 PMC8608337

[24] A. E. Ruf , W. K. Kremers , L. L. Chavez , V. I. Descalzi , L. G. Podesta , and F. G. Villamil , “Addition of Serum Sodium Into the MELD Score Predicts Waiting List Mortality Better Than MELD Alone,” Liver Transplantation 11 (2005): 336–343, 10.1002/lt.20329. 15719386

[25] A. K. Singal and P. S. Kamath , “Model for End‐Stage Liver Disease,” Journal of Clinical and Experimental Hepatology 3 (2013): 50–60, 10.1016/j.jceh.2012.11.002. 25755471 PMC3940492

[26] D. Dindo , N. Demartines , and P. A. Clavien , “Classification of Surgical Complications,” Annals of Surgery 240 (2004): 205–213, 10.1097/01.sla.0000133083.54934.ae. 15273542 PMC1360123

[27] A. Aloun , S. Akbulut , I. U. Garzali , et al., “Effect of Ursodeoxycholic Acid on Liver Regeneration Capacity After Living Donor Hepatectomy: A Prospective, Randomized, Double‐Blind Clinical Trial,” European Review for Medical and Pharmacological Sciences 27 (2023): 999–1006, 10.26355/eurrev_202302_31194. 36808345

[28] E. Sparrelid , S. Gilg , and T. M. van Gulik , “Systematic Review of MARS Treatment in Post‐Hepatectomy Liver Failure,” HPB 22 (2020): 950–960, 10.1016/j.hpb.2020.03.013. 32249030

[29] S. Gilg , E. Sparrelid , L. Saraste , et al., “The Molecular Adsorbent Recirculating System in Posthepatectomy Liver Failure: Results From a Prospective Phase I Study,” Hepatology Communications 2 (2018): 445–454, 10.1002/hep4.1167. 29619422 PMC5880195

[30] D. Inderbitzin , B. Muggli , A. Ringger , et al., “Molecular Absorbent Recirculating System for the Treatment of Acute Liver Failure in Surgical Patients,” Journal of Gastrointestinal Surgery 9 (2005): 1155–1162, 10.1016/j.gassur.2005.07.026. 16269387

[31] R. Kellersmann , H. J. Gassel , C. Bühler , et al., “Application of Molecular Adsorbent Recirculating System in Patients With Severe Liver Failure After Hepatic Resection or Transplantation: Initial Single‐Centre Experiences,” Liver 22 (2002): 56–58, 10.1034/j.1600-0676.2002.00011.x. 12220306

[32] M. P. Van De Kerkhove , K. P. De Jong , A. M. Rijken , A. C. J. M. De Pont , and T. M. Van Gulik , “MARS Treatment in Posthepatectomy Liver Failure,” Liver International 23 (2003): 44–51, 10.1034/j.1478-3231.23.s.3.2.x. 12950961

[33] A. Engin , “Bile Acid Toxicity and Protein Kinases,” in Protein Kinase‐Mediated Decisions Between Life and Death [Internet], ed. A. B. Engin and A. Engin (Springer International Publishing, 2021), 229–258, 10.1007/978-3-030-49844-3_9. 33539018

[34] C. Gräfe , H. Graf , V. Wustrow , et al., “Correlation of Bilirubin and Toxic Bile Acids in Critically Ill Patients With Cholestatic Liver Dysfunction and Adsorber Application,” Scientific Reports 14 (2024): 21762, 10.1038/s41598-024-72676-6. 39294181 PMC11411055

[35] M. J. Perez and O. Briz , “Bile‐Acid‐Induced Cell Injury and Protection,” World Journal of Gastroenterology 15 (2009): 1677–1689, 10.3748/wjg.15.1677. 19360911 PMC2668773

[36] T. Vasavan , E. Ferraro , E. Ibrahim , P. Dixon , J. Gorelik , and C. Williamson , “Heart and Bile Acids—Clinical Consequences of Altered Bile Acid Metabolism,” Biochimica et Biophysica Acta (BBA)—Molecular Basis of Disease 1864 (2018): 1345–1355, 10.1016/j.bbadis.2017.12.039. 29317337

