# Supplementary material for: Extracorporeal Albumin Dialysis (OPAL) as Novel Therapeutic Bridging Option in Posthepatectomy Liver Failure
Source: Artif Organs. 2026 Jun 14;50(9):1254–63. doi: 10.1111/aor.70187 (PMC13525224; doi:10.1111/aor.70187)
Supplement: Supplementary file 1 [file AOR-50-1254-s001.docx]

| **Pt** | **~** | **0h** | **Δ** | **24h** | **Δ** | **48h** | **Δ** | **72h** | **Δ** |
| --- | --- | --- | --- | --- | --- | --- | --- | --- | --- |
| 1 | 15.08 | 20.33 | 5.25 | - | - | - | - | - | - |
| 2 | - | 9.48 | - | 11.34 | - | 11.63 | - | 12.26 | - |
| 3 | 16.45 | 15.86 | -0.59 | - | - | - | - | - | - |
| 4 | 14.39 | 18.48 | 4.09 | - | - | - | - | - | - |
| 5 | - | - | - | - | - | - | - | - | - |
| 6 | - | - | - | - | - | - | - | - | - |
| 7 | 5.15 | - | - | - | - | - | - | - | - |
| 8 | - | - | - | - | - | - | - | - | - |
| 9 | 10.39 | 9.2 | -1.78 | - | - | - | - | - | - |
| 10 | 6.2 | 8.29 | 2.09 | 8.03 | 1.83 | 8.97 | 2.77 | 9.05 | 2.85 |
| 11 | 10.98 | 9.2 | -1.78 | 10.71 | -0.27 | - | - | - | - |
| 12 | 5.72 | 4.62 | -1.1 | 8.92 | 3.2 | 12.03 | 6.31 | 12.32 | 6.6 |
| 13 | 15.42 | 10.77 | -4.65 | 14.55 | -0.87 | - | - | - | - |
| 14 | 7.25 | - | - | - | - | - | - | - | - |
| 15 | 6.96 | 5.4 | -1.56 | 6.65 | -0.31 | 6.96 | 0 | 7.14 | 0.18 |
| 16 | - | - | - | - | - | - | - | - | - |
| 17 | - | 5.61 | - | 8.64 | - | 9.5 | - | 10.55 | - |
| 18 | - | - | - | - | - | - | - | - | - |
|  | 10.39 (6.2-15.08) | 9.48 (5.61-15.86) | -0.89  (-1.67-3.09) | 8.92 (8.03-11.34) | -0.27  (-0.59-2.52) | 9.5  (7.97-11.83) | 2.77  (0-6.31) | 10.55 (8.1-12.29) | 2.85 (0.18-6.6) |

**Supplemental Table 1 – Course of Bilirubin after discontinuation of OPAL.** h hours, *Pt* patient. ~ indicates the last laboratory testing during OPAL running time, 0h indicates the first laboratory testing after discontinuation of OPAL, Δ indicates the change compared to the value during OPAL running time, – indicates the absence of data because patients deceased, are still under OPAL treatment at the time of assessment or laboratory values are missing at the respective time points.
